# Translation and validation of a patient satisfaction survey: The isiXhosa version

**DOI:** 10.4102/phcfm.v5i1.515

**Published:** 2013-08-14

**Authors:** Tania Steyl, Julie Phillips

**Affiliations:** 1 Department of Physiotherapy, University of the Western Cape, South Africa

## Abstract

**Background:**

Although the use of surveys has been supported for assessing understanding of health care service quality, it could also be argued that their main function is to quantify perceptions. The importance of assessing patient satisfaction in individuals’ own language has been highlighted in research. However, important culture-specific differences can be revealed during the adaptation process of a scale, and if not attended to can influence the validity of the scale.

**Objectives:**

The aim of this study was to assess the validity and reliability of the isiXhosa version of the Patient Survey for Quality of Care (PSQC) in primary health care (PHC) facilities in a selected district in the Western Cape, South Africa.

**Method:**

The PSQC was translated into isiXhosa by two independant translators and the translated back into English by a third translator. All three translators reviewed the back translation. Face and content validity of the scale were assessed. Fifteen isiXhosa-speaking clients with type 2 diabetes mellitus who had a mean age of 62.27 years (SD 10.33) and came from a randomly selected community health centre participated in the test-retest reliability.

**Results:**

Internal consistency of the scale was good (Cronbach alpha 0.70). Alpha values of individual items relating to quality of care as well as items flagged for inferior service quality were between 0.772 and 1.000, indicating good to high internal consistency.

**Conclusion:**

Results of this study indicated that the isiXhosa version of the PSQC was as reliable as the English version. It can be implemented at PHC level to assess isiXhosa-speaking patients’ satisfaction with health care services.

## Abstrait

**Traduction et validation de l'Enquête sur les patients de la qualité des soins pour les clients ayant un diabète de type 2: la version isiXhosa**

**Contexte:**

Bien que l'utilisation d'enquêtes ont été pris en charge pour l’évaluation de la compréhension de la qualité des services de soins de santé, il pourrait aussi faire valoir que leur principale fonction est de quantifier les perceptions. L'importance de l’évaluation de la satisfaction du patient dans sa propre langue individus a été mis en évidence dans la recherche. Toutefois, des différences importantes spécifiques à la culture peuvent être révélées au cours du processus d'adaptation de l’échelle, et si elle n'est pas assisté d’, peuvent influer sur la validité de l’échelle.

**Objectifs:**

Le but de cette étude était d’évaluer la validité et la fiabilité de la version xhosa de l'enquête du patient pour la qualité des soins dans les établissements de soins primaires dans un quartier choisi dans le Western Cape, Afrique du Sud.

**Méthode:**

L'enquête du patient pour la qualité des soins a été traduite en xhosa par deux traducteurs indépendants et le dos traduits en anglais par un traducteur tiers. Tous les trois traducteurs revus la traduction en arrière. La validité apparente et la teneur de l’échelle ont été évalués. Quinze isiXhosa parler avec les clients diabète sucré de type 2 avec un âge moyen de 62,27 années (SD 10.33) à partir d'un centre de santé communautaire choisis au hasard, ont participé au test-retest fiabilité.

**Résultats:**

La cohérence interne de l’échelle était bon (Cronbach alpha = 0,70). Les valeurs alpha des différents articles relatifs à la qualité des soins ainsi que des drapeaux pour la qualité de service inférieure se situaient entre 0,772 et 1,000, ce qui indique une bonne cohérence interne de haut.

**Conclusion:**

Les résultats de cette étude ont indiqué que la version xhosa de l'enquête du patient pour la qualité des soins était aussi fiable que la version anglaise. Il peut être mis en œuvre au niveau des soins de santé primaires afin d’évaluer la satisfaction des patients de langue xhosa l’égard des services de soins de santé.

## Introduction

### Problem statement

Importance of assessing patient satisfaction in individuals’ own language has been highlighted in research.^1, 2^ The majority of established patient satisfaction scales stem from developed countries and have not been validated in the South African context. As most patient satisfaction scales are published in English, they need to be translated into isiXhosa, given that isiXhosa cultural background and language differ from those of English-speaking countries.

#### Key focus

Although the use of surveys has been supported for assessing understanding of health care service quality,^1, 2, 3, 4^ it could also be argued that their main function is to quantify perceptions. The Patient Survey for Quality of Care (PSQC) was translated into isiXhosa and assessed for face and content validity.

#### Background

Measuring patient satisfaction with health care services has been recognised as an important component in assessing service quality.^1, 2, 3, 4^ Patient perspectives about level of care can result in feedback which is useful for promoting higher-quality standards of patient care.^5^ Although patient satisfaction surveys are increasingly endorsed as a means of understanding health care service quality,^6^ for various reasons it could be argued that their function should include a measurement to quantify perceptions.

These surveys are inexpensive and easy to complete; they highlight aspects of care that need improvement in the health care setting^1, 7^ and provide concrete data to allow management to make the necessary changes to improve quality of service at their facility.^6^ The South African Department of Health's policy on quality in health care^8^ reiterates that public services need to respond to clients’ requests and expectations. Feedback from patient satisfaction surveys could assist with improved prioritisation and allocation of resources, and will also serve as a platform for providing better services to citizens.^4^

Researchers argue that good patient satisfaction scales must fulfill at least three requirements^9^: reliability, validity and transferability. Although many generic measurement scales exist to assess patient satisfaction with health care services,^1, 2, 3, 4, 5^ their use in clinical settings in Africa and South Africa specifically may be limited. Following a review of the literature pertaining to patient satisfaction scales it was concluded that a brief, easy to complete questionnaire suitable for community-based settings would be most appropriate. The ten-item scale of Woodward *et al*.^10^ was deemed most appropriate.

Generic scales are not necessarily culturally sensitive, and thus may need to be adapted for a specific context. Populations and cultural subgroups within populations differ in language, lifestyle, customs and perceptions of life.^11, 12^ For these reasons it is not advisable to directly administer existing and previously validated scales in different countries, cultures and language groups. The concern of misinterpretation of questions leading to compromised validity of responses has been highlighted.^11, 13, 14^

South Africa is known for its multicultural population. As stated earlier, languages are highly associated with cultures. It is important to address cultural differences when translating assessment tools, as the translated version may express the experiences of the patient differently from what was expressed in the original version.^15^

The isiXhosa-speaking population is the second largest ethnic group in South Africa, with approximately 1.1 million isiXhosa-speaking people living in the Western Cape, South Africa.^16, 17^ After Zulu, IsiXhosa is the second most widely spoken African language in South Africa, and nearly a quarter (23.7%) of the Western Cape's population speaks isiXhosa.^16^

Most established patient satisfaction scales stem from developed countries and have not been validated in the South African context. As most patient satisfaction scales are published in English, they need to be translated into isiXhosa, since the isiXhosa cultural background and language are different from those of English-speaking countries.

#### Objectives

Against this background, the main purpose of this study was to assess the validity and reliability of the isiXhosa version of the PSQC in a primary health care (PHC) facility in a selected district of the Western Cape, South Africa.

#### Contribution to field

The scarcity of published data regarding instruments translated into isiXhosa highlights the need for valid and reliable instruments with which to determine patient satisfaction with services provided at PHC facilities in an urban isiXhosa-speaking population. A validated, reliable isiXhosa scale could be used to assess isiXhosa-speaking patients’ satisfaction with health care provision at different PHC facilities in South Africa.

## Research method and design

### Materials

The PSQC^10^ contains ten items relating to quality of care, namely: 1) waiting time for an appointment; 2) waiting time in the clinic waiting room; 3) preparatory instructions given; 4) ease of obtaining information from staff; 5) clarity of information provided; 6) interpersonal manner of the staff; 7) attention paid to safety of the client and security of their belongings; 8) attention paid to privacy; 9) instructions upon leaving; and 10) the client's perception of overall quality of care.

Each item had five response choices, namely poor, fair, good, very good and excellent. An additional three items which were considered to be flags of inferior service quality were included, which asked whether clients were asked to leave the facility before they felt ready to do so; whether their health had deteriorated or they had required another visit to the clinic shortly after the service was provided; and whether they would recommend the service to a family member or friend who needed such a service. Each of these three items had two response choices, namely yes or no. Lastly an open-ended question was included which asked about suggested improvements, if needed.

### Setting

Data were collected at a PHC setting in the Cape Metro District of the Western Cape. The randomly selected community health centre (CHC), situated in the Cape Flats, is one of three CHCs serving mainly isiXhosa-speaking clients. The CHC is situated in a partially informal township with a population of approximately 420 000 people.

### Design

The study incorporated a repeated-measures study design.

### Procedure

#### Forward-and-back translation process

An independant freelance Xhosa translator and a native Xhosa-speaking health professional were consulted for the isiXhosa forward translation of the PSQC scale. The translators were not informed of the study purpose, and were requested to review and compare the translated PSQC scale with the original PSQC scale. The translated PSQC scale was then sent to a professional Xhosa translator to perform translation of the isiXhosa version of the PSQC back into English. The original English version of the PSQC was not given to the back translator as a reference. The isiXhosa version of the PSQC scale then underwent professional editing and the pre-final version was produced for testing of psychometric properties, namely face and content validity, and test-retest reliability.

### Analyses

#### Quantitative data

Data were extracted, entered and analysed using the Statistical Package for Social Sciences version 20. Internal consistency of individual items of the PSQC scale was assessed using the Cronbach alpha coefficient, with alpha values above 0.70 considered good.^18^ The alpha coefficient introduced in Cronbach^19^ is a statistic used often in empirical research involving different items. The reliability of a scale is assessed by Cronbach's alpha coefficient by determining internal consistency of the scale or the average correlation of items within the scale.^18^ This coefficient ranges in value from 0 to 1, and the higher the score, the more reliable the scale. An acceptable reliability coefficient of 0.7 has been indicated, although lower levels are often used in the literature.^17^

The one-sample *t*-test and intraclass co-efficient (ICC) with a 95% confidence interval (CI) were used to assess test-retest reliability (stability) of the questionnaire. An ICC of above 0.81 was considered almost perfect agreement; 0.61–0.80 indicated substantial agreement; 0.41–0.60 indicated moderate agreement; 0.21–0.40 indicated fair agreement; and below 0.20 poor agreement.^19^

#### Qualitative data

The responses collected during the face and content validity testing were coded and analysed qualitatively. Audiorecorded data from the informal discussions were transcribed verbatim by a person fluent in both isiXhosa and English. The transcript was compared with the field notes taken by the research assistant. The isiXhosa transcript was then translated back into English by another person that speaks both English and isiXhosa. To assist with validity of the translation process it is of utmost importance that the back translation is done by a different person from the translator who does the forward translation.^20^

## Results

### Face and content validity

The group which participated reported that the items were clear and appropriate to assess their satisfaction with quality of services of the CHC. In addition the questions were easy to understand, they understood what was meant by each question, and they were given enough time to complete the questionnaire. No changes were required. Examination of the scale by the researcher (whilst entering data for analysis) showed that the completeness of item responses was excellent, as no missing data were found. This could also be an indicator that participants understood the questions.

### Reliability

Fifteen participants, six male and nine female patients with a mean age of 62.27 years (SD 10.33), participated in the test-retest study. ICC was the measure used to determine the internal consistency of individual items of the scale; this ‘correlates’ each individual question with the total and gives a value between 0 and 1. A value of 0 indicates that none of the questions correlate well with the total, whilst a value of 1 indicates excellent correlation. Alpha values of 0.70 and above were considered good.^10^

The results showed that the internal consistency for all ten items relating to quality of care was high, exceeding 0.7 for all, as seen in Table 1. Internal consistency of the overall scale was good (Cronbach alpha 0.70). All three questions which were considered flags for inferior service quality had excellent correlation (ICC 1.000). None of the participants completed the last question regarding suggestions for improvement of the CHC's services.

**TABLE 1:** Test-retest scores per item of the PSQC.

Question	ICC	95% CI
1. Waiting time. How long you had to wait to get an appointment at this clinic	1.000	1.000–1.000
2. Waiting time. How long you had to wait in the clinic waiting room for your appointment	0.947	0.843–0.982
3. Instructions. How well the clinic staff prepared you on what to expect at the visit	0.932	0.798–0.977
4. Ease of getting information. Willingness of clinic staff to answer your questions	0.904	0.714–0.968
5. Information you were given. How clear and complete the explanations were on any information you were given regarding your diabetes	0.772	0.321–0.923
6. Concern and caring by clinic staff. Respect, friendliness and kindness	0.873	0.621–0.957
7. Safety and security. The provision for your safety and the security of your belongings	0.845	0.539–0.948
8. Privacy. How well your privacy was considered, e.g. with results of blood sugar tests, weight, etc.	0.839	0.520–0.946
9. Instructions upon leaving. How clearly and completely you were told what to do at home or until your next visit	0.788	0.368–0.929
10. Overall quality of care. How you evaluate services you received and the way you were treated	0.788	0.368–0.929

ICC, intraclass co-efficient; IC, confidence interval.

## Ethical considerations

Ethical approval was obtained from the relevant university authority of the University of the Western Cape, South Africa (clearance number 11/4/2), the Western Cape Department of Health and the facility manager of the selected CHC.

### Potential benefits and hazards

The study had no envisioned risks for participants. Benefits included development of a valid and reliable scale to determine the satisfaction of isiXhosa-speaking patients from an urban community with services provided at PHC level.

### Recruitment procedures

The sample size required for the study was based on an ICC of 0.9 and a maximum width of 0.23 for the 95% CI, based on previous studies.^21^ Every second patient attending the CHC on the day of data collection was randomly invited to participate in the study. A total of 25 patients was approached for participation and 15 (60%) agreed to do so. Subject recruitment criteria included:

- male and female patients aged 18 years and older;
- patients diagnosed with type 2 diabetes mellitus attending the Diabetes Clinic at a specific CHC in the Cape Metropolitan District of the Western Cape, South Africa; and
- who spoke and comprehended the isiXhosa language.

### Informed consent

The purpose of the study was clearly explained to all consenting participants using an information sheet. Signed written informed consent was obtained from all participants. The consent form, information sheet and questionnaire were available in isiXhosa. Participation in the study was voluntary and participants were informed of their right to withdraw from the study at any time without any consequences. Identification codes using numbers were used on data forms to ensure anonymity. Only the researcher and research assistant had the details of the participants. Information obtained from the informal discussions was handled with confidentiality, and participants in the informal discussion signed a form where they undertook not to disclose any information from it.

### Data protection

The researcher personally collected the completed questionnaires and informed the participants that they would be stored in a locked and secure place. All audiotapes of informal discussions were destroyed once they had been transcribed and documented according to themes. The transcribed data were also stored in a locked and safe place.

## Trustworthiness

### Reliability

Test-retest reliability (stability of responses to questions over time)^22^ was assessed. The questionnaire was administered to the participants at the selected CHC. To measure internal consistency and test-retest reliability of the isiXhosa version of the PSQC scale, participants were required to complete the questionnaire twice, at different time points. After completing the questionnaire for the first time, participants were requested to return to the CHC within two weeks to complete it for a second time.

### Validity

Validity is one of the most important criteria by which a quantitative instrument's adequacy is evaluated.^22^ The pre-final version of the isiXhosa translation of the PSQC scale was tested amongst the group of patients with type 2 diabetes mellitus to establish face and content validity, acceptability and comprehensibility. Content validity (whether items on the scale measure what they are supposed to measure)^22^ was judged by an expert in questionnaire development and Xhosa-speaking health care professionals working at the CHC. They were called on to analyse the items to see if they adequately represented the hypothetical content universe in the correct proportions.

The isiXhosa version of the PSQC scale was administered to participants with type 2 diabetes mellitus. The purpose was to ensure that the translated PSQC scale was understood in the local context and that the items measured what they were supposed to. Participants were asked to comment on their understanding of the instructions provided, ease of understanding the questions, time provided for completing the questionnaire, and ease of completing it. The comments made by the participants were collected and evaluated.

Criterion validity (which relates to how well the scale correlates with a ‘gold standard’ measure)^22^ could not be assessed. No published data regarding patient satisfaction with quality of care scales or instruments translated into isiXhosa were found; hence no there was no ‘gold standard’ against which validity of the isiXhosa version of the PSQC scale could be measured.

### Generalisability

Generalisability refers to the degree to which results of research can be generalised or transferred to other contexts or settings.^22^ The isiXhosa version of the scale was only tested for reliability and validity on an urban isiXhosa-speaking population with type 2 diabetes mellitus. The scale can therefore be used at any urban PHC setting to assess isiXhosa-speaking peoples’ satisfaction with health care services in South Africa.

## Discussion

### Outline of the results

The importance of assessing patient satisfaction in individuals’ own language has been highlighted, and this study aimed to assess reliability and validity of the translated isiXhosa version of the PSQC for isiXhosa-speaking patients in South Africa. Important culture-specific differences can be revealed during the adaptation process of a scale, and if not attended to can influence validity of the scale. Furthermore, an incongruous translation process could lead to inaccurately translated questionnaires which may not contain meanings equivalent to the original version.^23^ To this end researchers have highlighted that adaptation of scales for different cultures is a complex process and involves much more than just linguistic translation.^12^

To avoid misrepresentation of the isiXhosa version independent forward and back translators were asked to prepare the scale.^22^ The scale was also reviewed by all translators as well as an expert in questionnaire development and health care professionals working in PHC facilities. This process ensured that the translation process was acceptable and valid.

Thus a scale assessing outcomes should be stable at baseline, as it will be difficult to detect changes after an intervention. The test-retest reliability is a measure of the same results obtained with a scale on repeated administrations. In this study a 2-week time interval was used. This interval was chosen as it minimises possible memory effects and no change could occur as patients only visit the health care facilities once every three months. The test-retest reliability for this scale was 0.70.

### Practical implications

Given the high correlation of scores of individual items on the scale, the results confirmed that the isiXhosa version of the PSQC scale is a highly reliable and valid tool to determine patient satisfaction with services provided at PHC facilities in an urban isiXhosa-speaking population.

## Limitations of the study

The small sample size and one urban research site for isiXhosa-speaking patients with type 2 diabetes mellitus (as ethically approved for the study) limit transferability of the findings to the general population with type 2 diabetes mellitus.

### Recommendations

It is recommended that patients from rural areas should be included in the validity and reliability testing of the isiXhosa version of the PSQC scale.

## Conclusion

The isiXhosa version of the PSQC has good psychometric properties and may be considered ready for use to assess urban isiXhosa-speaking patients’ satisfaction with health care services at PHC level. This translated scale would also be useful for future research as no isiXhosa version of this scale existed before. Information gathered from future research could thus assist management of health care services to identify problem areas and continuously make changes to improve quality of care.
